# Case Report: Pyoderma gangrenosum masquerading as skin and soft tissue infection preceding myelodysplastic syndrome

**DOI:** 10.3389/fmed.2026.1792269

**Published:** 2026-04-14

**Authors:** Yan Shi, Bo Tang, Tao Qu, Shuangni Yu, Jun Feng, Xiao Chang, Xiaoting Wang

**Affiliations:** 1 Department of Intensive Care Unit, Peking Union Medical College Hospital, Peking Union Medical College and Chinese Academy of Medical Sciences, Beijing, China; 2 Department of Dermatology, National Clinical Research Center for Skin and Immune Diseases, Peking Union Medical College Hospital, Peking Union Medical College and Chinese Academy of Medical Sciences, Beijing, China; 3 Department of Pathology, Peking Union Medical College Hospital, Peking Union Medical College and Chinese Academy of Medical Sciences, Beijing, China; 4 Department of Hematology, Peking Union Medical College Hospital, Peking Union Medical College and Chinese Academy of Medical Sciences, Beijing, China; 5 Department of Orthopaedic Surgery, Peking Union Medical College Hospital, Peking Union Medical College and Chinese Academy of Medical Sciences, Beijing, China

**Keywords:** myelodysplastic syndrome, neutrophilic dermatoses, pathergy, pyoderma gangrenosum, skin and soft tissue infection

## Abstract

Pyoderma gangrenosum (PG) is a rare, complex, and severe inflammatory dermatosis categorized under the spectrum of neutrophilic dermatoses (NDs), pathologically characterized by sterile neutrophilic infiltration. It is frequently associated with systemic diseases and typically manifests concurrently with or after their diagnosis. We report a rare PG case of an elderly woman who presented 5 months prior to the onset of hematological malignancy (HM), and this unusual clinical presentation led to a misdiagnosis. The patient was initially suspected of having a lower-extremity skin and soft tissue infection (SSTI). Despite repeated debridement and broad-spectrum antibiotic therapy, her condition deteriorated progressively, eventually necessitating amputation. Negative microbial test results ruled out infection. Subsequently, the development of analogous lesions at the venipuncture sites prompted clinicians to reassess the overall clinical condition. Based on prominent neutrophilic infiltration revealed by skin biopsy pathology and the favorable response to glucocorticoid therapy, the initial lower extremity lesions were retrospectively diagnosed as PG. During the follow-up, the patient was diagnosed with myelodysplastic syndrome and was initiated on corresponding treatment. This unusual case enriches the understanding of the PG-HM association and underscores the importance of clinical vigilance and timely reassessment to optimize prognosis.

## Introduction

Neutrophilic dermatoses (NDs) are a group of rare, complex inflammatory disorders characterized by prominent neutrophilic infiltration within the skin in the absence of infection or true vasculitis (1, 2). As a heterogeneous spectrum of inflammatory conditions, NDs include Sweet’s syndrome (SS), pyoderma gangrenosum (PG), subcorneal pustular dermatosis (Sneddon–Wilkinson disease), and neutrophilic panniculitis (3, 4). NDs are frequently associated with various systemic diseases, predominantly inflammatory bowel disease (IBD), rheumatic disorders, and hematological malignancies (HMs), and they usually develop concurrently with or after these underlying conditions (5–7). In this study, we report an unusual case of an elderly woman who was initially suspected of having a skin and soft tissue infection (SSTI). She underwent multiple debridement procedures combined with broad-spectrum antibiotic therapy; however, her condition continued to deteriorate, eventually necessitating amputation. During hospitalization, similar ulcerative lesions developed at the venipuncture sites, prompting clinicians to conduct a comprehensive reassessment of the entire clinical context. A definitive diagnosis of PG was subsequently made, and myelodysplastic syndrome (MDS) was confirmed during follow-up in the following months. This case not only enriches the current understanding of the association between PG and underlying HMs but also provides a reference for optimizing the diagnostic strategies and therapeutic regimens for such patients, thereby facilitating the enhancement of clinical management of related disorders.

## Case presentation

We present the case of a 73-year-old woman who presented with erythema, swelling, and pain of the left lower leg, with no significant past medical history. After 10 days of topical antibiotic treatment, the patient developed progressive skin ulceration, exacerbated pain, and fever (body temperature: 38.3 °C), prompting transfer to our emergency department.

**Figure 1:**
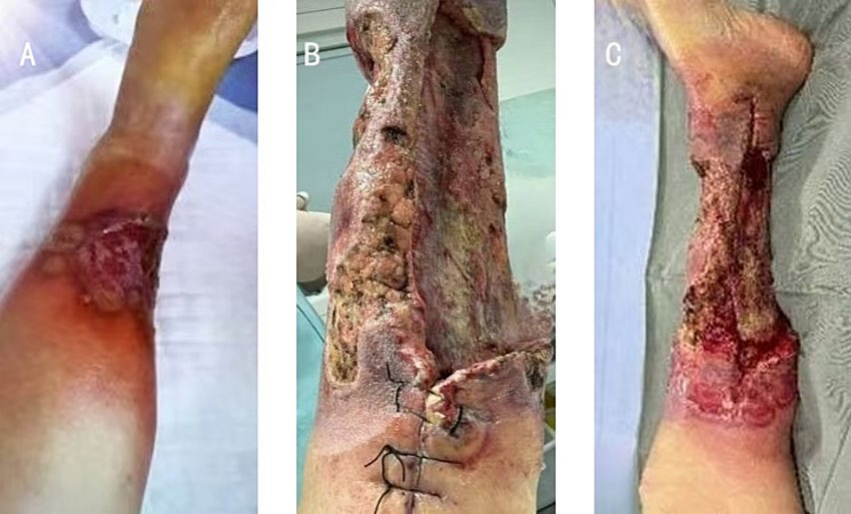
Progressive manifestations of necrosis on the lower extremity during hospitalization. **(A)** At admission, **(B)** before the third surgical debridement, and **(C)** before amputation surgery.

On admission, a physical examination revealed swelling of the left lower leg with extensive epidermal exfoliation on the medial and posterior aspects, forming a large ulcer. The irregular ulcer border was elevated and dusky-red (Figure 1A). No crepitus was noted on palpation of the swollen area. Lower extremity blood supply was normal, with intact tactile and pain sensation. Laboratory workup revealed a white blood cell (WBC) count of 5.18 × 10^9^/L with 4.41 × 10^9^/L neutrophils, accompanied by decreased hemoglobin (91 g/L) and platelet count (89 × 10^9^/L). Inflammatory markers were elevated: the erythrocyte sedimentation rate (ESR) was 78 mm/h, and the C-reactive protein (CRP) level was 135 mg/L. Aspartate aminotransferase, alanine aminotransferase, lactate dehydrogenase, creatine kinase, and myoglobin levels were within normal ranges.

A preliminary diagnosis of SSTI was made, and emergency debridement was performed on 9th June. Postoperatively, empirical therapy with piperacillin/tazobactam (4.5 g IV q6h) combined with linezolid (600 mg IV q12h) was administered for 1 week. Given negative acid-fast bacilli and Gram stains of tissue specimens, with no microbial growth on culture, antibiotics were de-escalated to ceftriaxone (1 g IV qd) and clindamycin (600 mg IV q12h). However, the patient developed recurrent fever (maximum temperature 38.5 °C) on postoperative day 5, with extension of leg necrosis, and a second extended debridement was performed on 18th June. Postoperative fever persisted (temperature fluctuating between 37.8 °C and 38.3 °C), and wound necrotic tissue cultures remained negative. On 21st June, she developed pancytopenia (WBC: 3.94 × 10^9^/L, hemoglobin: 85 g/L, and platelets: 43 × 10^9^/L), which was initially attributed to poorly controlled infection. Concomitant isolation of *Candida albicans* and *Enterococcus faecium* from wound swab cultures led to a change in antibiotics to linezolid (600 mg IV q12h), fluconazole (200 mg IV q12h), and piperacillin/tazobactam (4.5 g IV q6h); however, no clinical improvement was observed, and a third debridement of necrotic tissue was performed on 1st July (Figure 1B). A histopathological examination of tissue specimens from all three surgeries revealed only fibroadipose tissue with extensive necrosis. Immunohistochemistry (IHC) showed CD20 (−) and a Ki-67 proliferation index of 2%, with no evidence of lymphoma. Subsequently, the patient became progressively debilitated and bedridden, with worsening lower limb ulceration (Figure 1C). Necrotizing fasciitis could not be ruled out, and she underwent above-knee amputation under general anesthesia on 13th July, followed by transfer to the intensive care unit.

Postoperatively, ventilator-assisted ventilation was initiated, and low-dose norepinephrine (0.1 μg/kg/min) was required to maintain blood pressure. Empirical anti-infective therapy with cefepime (1 g IV q12h), tigecycline (loading dose: 100 mg IV and maintenance dose: 50 mg IV q12h), and fluconazole (200 mg IV q12h) was administered concurrently. Despite amputation, the patient continued to have a fever (temperature: 37.8 °C–38.7 °C) accompanied by progressive pancytopenia (WBC: 1.63 × 10^9^/L, hemoglobin: 69 g/L, and platelets: 31 × 10^9^/L), as well as elevated CRP (184 mg/L), ferritin (4,173 ng/mL), and Epstein–Barr virus load (17,018 copies/mL). No pathogenic microorganisms were identified on special stains, such as periodic acid–Schiff, Gomori methenamine silver, and fungal fluorescence, cultures, or metagenomic next-generation sequencing (mNGS) of residual limb tissue, including bacteria, fungi, *Mycobacterium tuberculosis*, non-tuberculous mycobacteria, viruses, and parasites. Given persistently negative microbial findings and a lack of response to broad-spectrum antibiotics, an infectious process was excluded as the etiology of the overall clinical picture. Bone marrow aspiration was performed on 20th July, revealing significant hypoplasia of hematopoietic tissue with a roughly normal myeloid-to-erythroid ratio; no megakaryocytes or hemophagocytosis were observed. Additional laboratory tests, including antinuclear antibody titer, vasculitis antibodies, and rheumatoid factor, were negative, excluding autoimmune diseases. Serum protein electrophoresis and immunoelectrophoresis did not reveal monoclonality.

**Figure 2:**
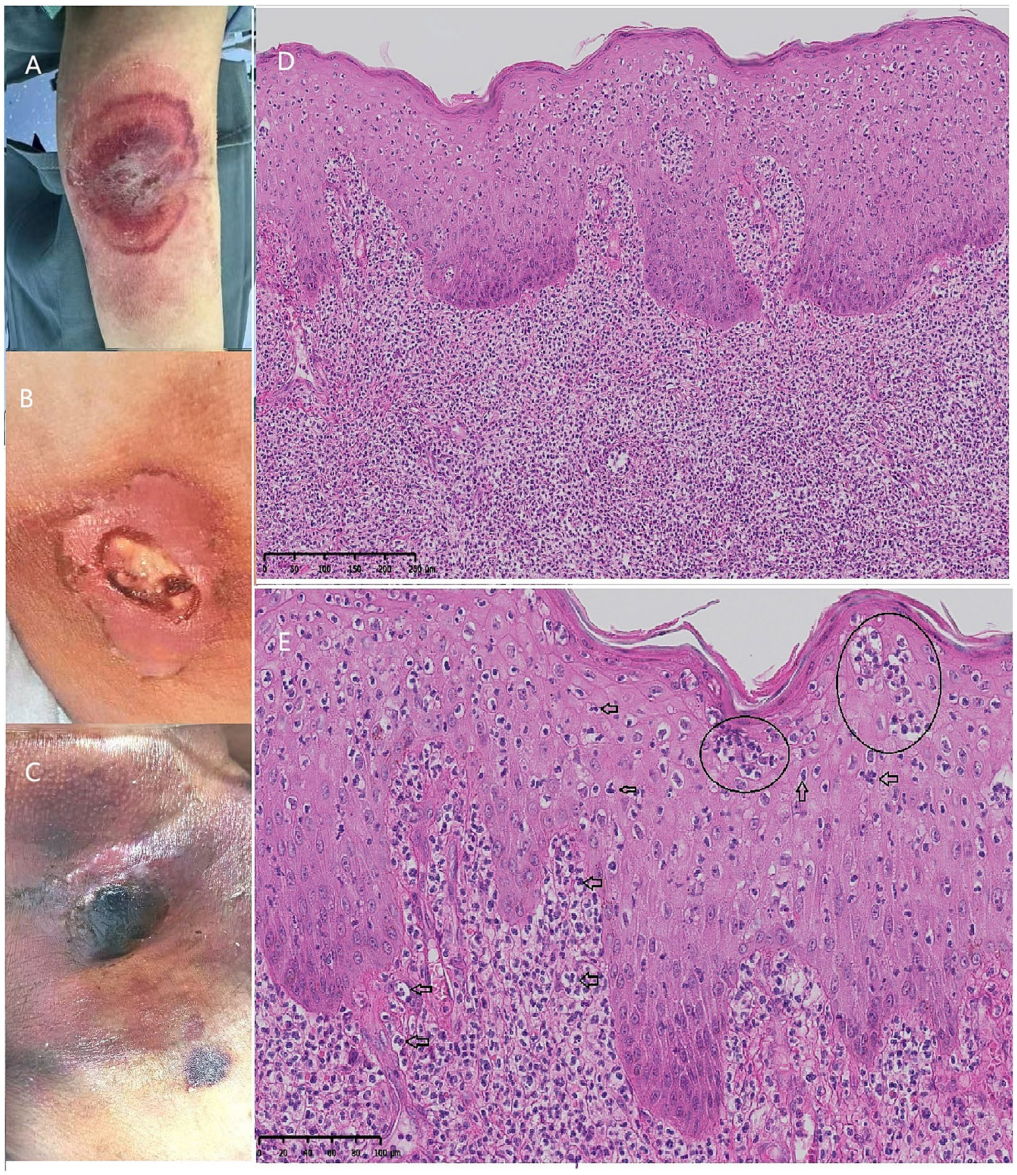
Clinical manifestations and histopathological findings of cutaneous lesions at acupuncture sites. **(A)** The initial lesion presented as a poorly defined, large erythematous plaque on the left forearm, with a small suppurative bulla in the center surrounded by a bright erythematous halo. **(B)** It progressed to a large ulcer subsequently. **(C)** A painful violaceous nodule was noted at the internal jugular vein acupuncture site, surrounded by multiple ecchymotic plaques. **(D)** Histopathologic examination revealed dense neutrophilic infiltration in the epidermis and papillary dermis, extending inferiorly into the deep dermis (hematoxylin and eosin [H&E], ×10 magnification). **(E)** High-power magnification showed neutrophilic aggregates and microabscesses (circles), as well as neutrophils in the epidermis and superficial dermis (arrows) (H&E, ×200 magnification).

Meanwhile, skin lesions similar to the initial leg lesion appeared at the peripheral vein and central vein puncture sites. The left forearm presented with a large, poorly demarcated erythematous plaque with a small suppurative bulla in the center, surrounded by a bright erythematous halo, which subsequently progressed to a superficial ulcer (Figures 2A,B). At the internal jugular vein puncture site, a painful violaceous nodule appeared, accompanied by multiple hemorrhagic vesicles (Figure 2C). The patient was consulted and evaluated by the dermatology department, and a skin biopsy was performed on the lesions. Histopathological examination revealed diffuse neutrophilic infiltration with abundant nuclear dust in the epidermis and superficial dermis, forming microabscesses. No vasculitis was identified. The lesion extended inferiorly into the deep dermis, and the subcutaneous adipose tissue was not significantly affected (Figures 2D,E). Microbiological examinations for bacteria, mycobacteria, and fungi were negative. Based on these pathological findings and cutaneous manifestations (painful hemorrhagic vesicles with inflammatory borders rapidly progressing to superficial ulcers), a diagnosis of ND was made, with a high suspicion for bullous PG. The patient was initiated on intravenous methylprednisolone 40 mg daily (equivalent to prednisolone 1 mg/kg/day), with wound care using collagenase and hyperabsorbent dressings.

Given the known association between PG and HMs, a repeat bone marrow aspiration was performed for chromosomal and genetic analysis, which revealed a *U2AF1* mutation; the CD38 (Csp8) expression rate was 77%, and the deletion rate of the chromosome 20 probe (D20S108) was 54%. Additionally, we requested pathological re-evaluation of the stump tissue to specifically rule out local infiltration of HMs. A histopathological examination revealed extensive necrosis of the skin, subcutaneous tissue, and striated muscle; no abnormalities were noted in deep striated muscle or bone tissue, and no vasculitic changes were observed. Prominent neutrophilic infiltration was identified in the skin and subcutaneous soft tissue surrounding the necrotic area, with focal aggregates of monocytoid cells. IHC staining of these monocytoid cells showed positivity for myeloperoxidase (MPO) and CD15, with a high proliferation index (Ki-67, 90%), whereas the remaining markers (CD2, CD3, CD5, CD7, CD8, CD20, CD21, CD30, CD34, CD56, CD68, CD99, CD117, CD123, and granzyme B) were negative. Fluorescence *in situ* hybridization (FISH) testing for myeloid neoplasms was negative for the RUNX1: RUNX1T1 (AML1: ETO) fusion gene [t(8;21)], and no abnormalities were detected in KMT2A (MLL) or CBFB genes. These findings do not support local infiltration by HMs.

## Outcome and follow-up

**Figure 3:**
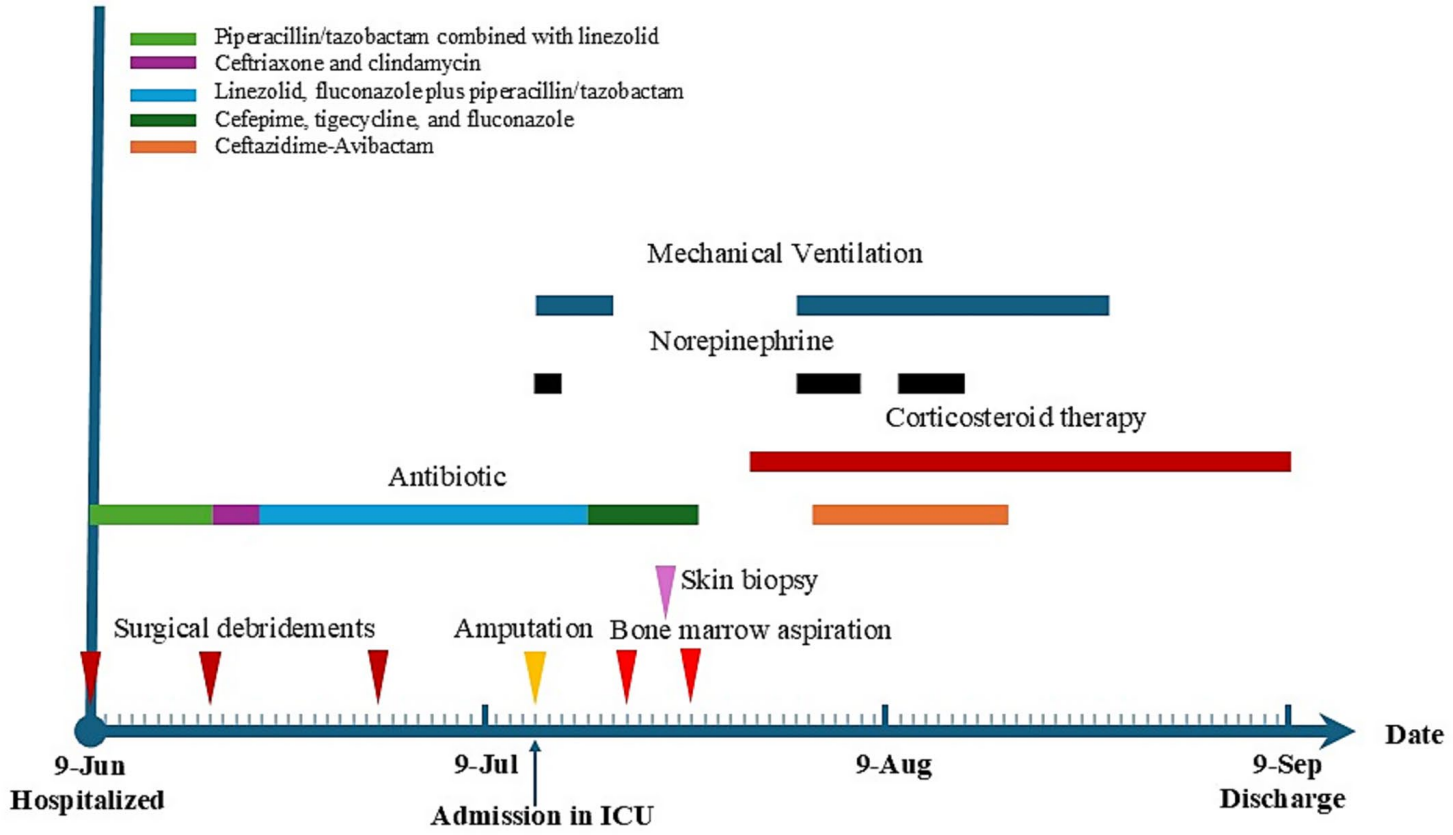
Timeline and treatment course for the patient.

Following the initiation of systemic corticosteroid (CS) therapy, the patient achieved an excellent initial response, both clinically (resolution of fever, pain, and cutaneous lesions) and laboratory-wise (a drastic reduction in CRP to 55 mg/L only 4 days after CS treatment initiation). Cutaneous lesions resolved completely 2 weeks later, and methylprednisolone was switched to oral prednisolone with a tapering regimen of 5 mg per week. The patient received supportive care for pancytopenia, including intermittent blood transfusion, granulocyte colony-stimulating factor, and thrombopoietin. Concurrently, ventilator-associated pneumonia and shock were actively managed. Three months after hospitalization, the patient was transferred to a rehabilitation hospital for continued follow-up (Figure 3).

**Figure 4:**
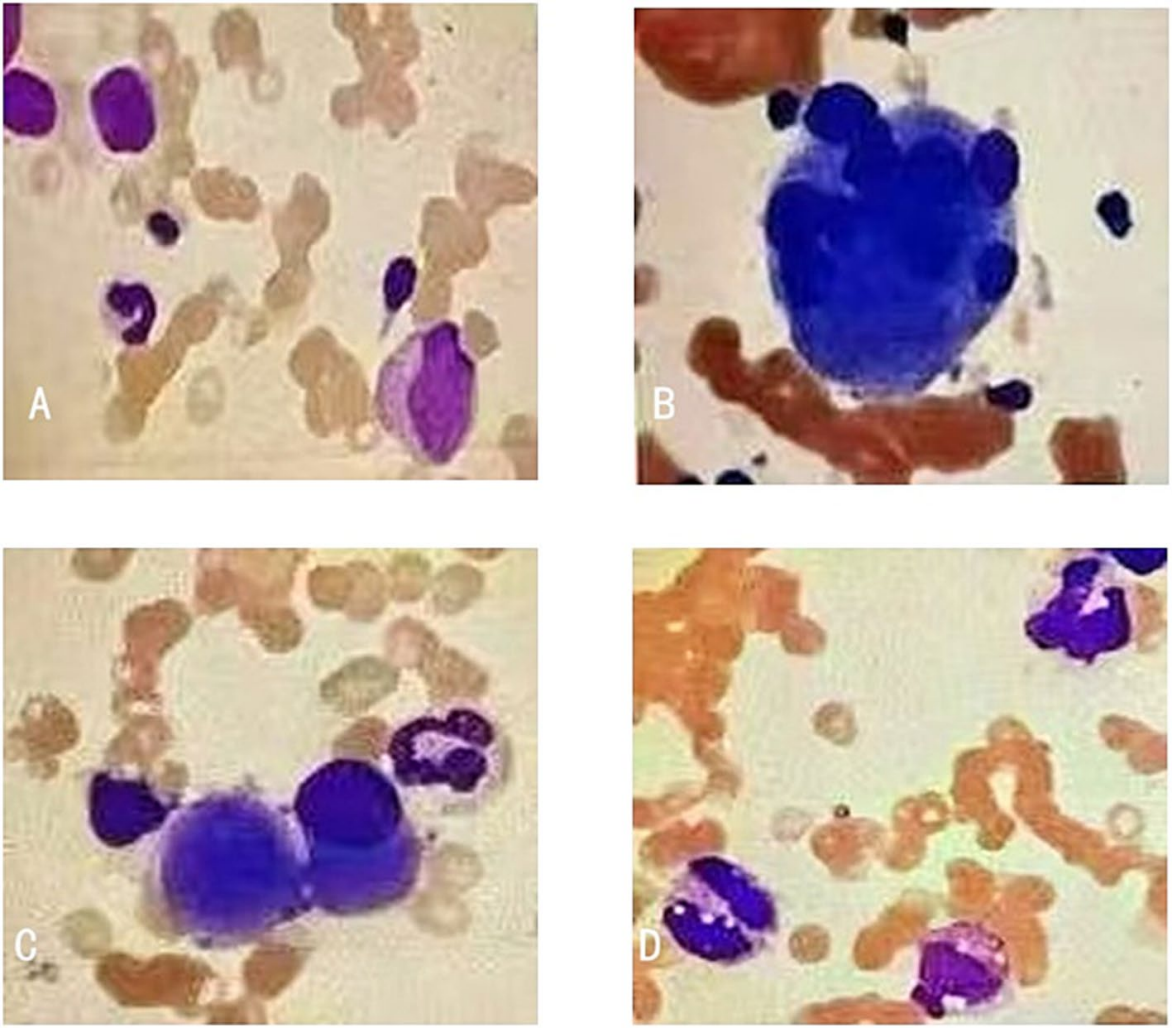
Morphological findings of bone marrow aspirate showed dyshematopoiesis. **(A)** Myeloblasts, **(B)** multinucleated megakaryocytes, **(C)** micromegakaryocytes, and **(D)** granulocytes with Pelger–Huët anomaly and binucleated granulocytes (×800 magnification).

By integrating clinical, anamnestic, laboratory, pathological, and treatment response data, this case fulfills the PARACELSUS scoring criteria (≥10 points) for the diagnosis of PG (8) and achieved a total score of 16 points: 8 points for major criteria (rapid progression and exclusion of other etiologies; 4 points each) and 8 points for minor criteria (pathergy reaction, associated systemic disease, consistent histopathology, and rapid response to treatment; 2 points each). Ultimately, the ulcers involving the upper and lower extremities and the neck were diagnosed as PG, complicated by an underlying hematological disorder suspicious for MDS. Two months later, when the prednisolone dosage was tapered to 5 mg daily, cutaneous lesions recurred; symptoms improved 1 week after increasing the prednisolone dose to 0.5 mg/kg/day. Considering that ND recurrence might be associated with the progression of the hematological disorder, a repeat bone marrow aspiration revealed bone marrow hypoplasia and dysplasia: myeloblasts accounted for 5% of the myeloid series, with nuclear malformation and binuclear changes observed in some granulocytes; micromegakaryocytes and multinucleated megakaryocytes were also frequently detected (Figure 4). MDS was comprehensively diagnosed based on bone marrow morphological findings, molecular cytogenetic abnormalities, and persistent pancytopenia. Considering the patient’s age and risk stratification, she was initiated on thalidomide and azacitidine therapy.

## Discussion

This report elaborates on the intricate diagnostic and therapeutic processes of a PG case, which manifested 5 months prior to the onset of the underlying hematological disorder and initially presented with features mimicking an SSTI, resulting in a significant diagnostic delay. The majority of published studies have focused on NDs triggered as a paraneoplastic syndrome, whereas reports describing NDs that precede the onset of the underlying disease remain relatively scarce. The unique chronological sequence of disease onset in this case expands the clinical spectrum of PG complicated by MDS and provides a valuable reference for optimizing the clinical management of similar cases.

PG is a rare, complex, and severe inflammatory dermatosis categorized within the spectrum of NDs. The estimated incidence of PG is approximately six cases per million individuals annually, occurring at any age but most commonly between 40 and 50 years, with a slight female predominance (9, 10). Since its original description by Brunsting et al. (11), the clinical spectrum of PG has been further delineated. Four major variants are currently recognized: classical ulcerative PG (the most common form), pustular PG, bullous PG, and granulomatous PG (12). Three diagnostic criteria—those proposed by Su et al. (13), the Delphi consensus (14), and the PARACELSUS score (8)—have been compared for their diagnostic accuracy in PG; PARACELSUS accurately scored 89% of PG patients, followed by the Delphi consensus and Su et al., which each assessed 74% of patients with PG (15). Our case fulfills all three widely used diagnostic criteria for PG, and its complex clinical course provides valuable insights for clinicians.

According to Weenig et al. (16), PG is a diagnosis of exclusion that includes infections (bacterial, viral, fungal, and protozoal), systemic vasculitis and vasculopathies, vascular disorders, and neoplastic and exogenous tissue injury. In the present case, no microorganisms were identified via tissue staining, culture, or even mNGS. Furthermore, the lack of clinical response to broad-spectrum antibiotic therapy effectively excludes infection as the cause of the entire clinical course. We also rigorously excluded immune-related and neoplastic mimics, particularly leukemia cutis (LC). Acute myeloid leukemia (AML) may uncommonly present as LC or neoplastic leukocytic skin infiltration with diverse cutaneous manifestations that render clinical distinction from other skin lesions challenging (17). Histologically, skin infiltrates in LC consist of malignant immature myeloblasts, and an IHC analysis demonstrates clonality of the abnormal cells (17, 18). In contrast, the histopathological examination of our patient’s skin lesions revealed predominantly mature neutrophil infiltration, with no significant evidence of abnormal cellular clonality on IHC. FISH testing for rearrangements or abnormalities in the AML1-ETO, CBFB, and MLL genes—commonly associated with AML—yielded negative results, thereby excluding LC as a diagnostic possibility.

Although both PG and SS fall within the spectrum of NDs, their clinical presentations differ substantially. SS typically manifests as painful, erythematous plaques and nodules, whereas subcutaneous SS is a rare variant characterized by deep subcutaneous nodules with normal or mildly erythematous overlying skin. Ulceration is typically absent in both forms. Histopathologically, classic SS involves the dermis, while the subcutaneous variant affects the subcutaneous fat (19). In contrast, classic ulcerative PG is characterized by painful, rapidly progressive necrosis leading to central ulceration with violaceous, undermined borders. Ulcerations may extend deeply, potentially exposing muscle and tendons (20, 21). Approximately 50% of PG cases are associated with underlying systemic disorders, including IBD, rheumatoid arthritis, non-Hodgkin’s lymphoma, and myeloproliferative neoplasms (9, 22, 23). Conversely, SS is more frequently associated with HMs, particularly MDS (19). Moreover, pathergy is observed almost exclusively in PG, representing another crucial distinguishing feature between the two disorders.

Pathergy is defined as the induction or exacerbation of PG-like lesions, including painful ulcers, pustules, and nodules, at sites of minor trauma, such as venipuncture, scratches, or surgical incisions. This characteristic manifestation occurs in approximately one-third of PG cases (24). Recognizing pathergy carries crucial clinical significance: surgical debridement can exacerbate the initial pathogenic process, inducing or accelerating the formation of new ulcers and tissue necrosis (25, 26). As illustrated in this case, repeated debridement procedures inadvertently exacerbated disease progression, ultimately necessitating surgical amputation. This outcome underscores the importance of early diagnosis and the avoidance of inappropriate surgical interventions.

Notably, PG typically occurs concurrently with or after the diagnosis of HMs, with only a rare subset of NDs preceding them (9, 22, 24). In a systematic review of MDS complicated with NDs, only one of eight cases reported an ND diagnosis prior to MDS (27). Notably, our patient exhibited the longest reported interval to date—5 months—between ND onset and the diagnosis of the underlying hematological disorder, which contributed substantially to the diagnostic delay. We provide a detailed account of the tortuous diagnostic process and treatment adjustments, offering practical clinical insights for identifying similar atypical cases.

PG carries a poor prognosis and is associated with high mortality (9, 21, 23). Additionally, when PG occurs alongside HMs, such as AML, approximately 75% of patients die from the underlying hemopathy within the first year (9, 23). Chadli et al. (28) reported a case of a young woman who presented with PG 3 weeks before AML onset. Although intravenous methylprednisolone (1 mg/kg/day) was administered, resulting in rapid resolution of PG, the patient died shortly afterward. Postmortem results of bone marrow aspirate revealed AML, with IHC analysis of the skin lesions confirming the clonality of neutrophils derived from the leukemic clone. Thus, persistent efforts should be made to identify underlying disorders in all PG cases, given that early diagnosis and targeted treatment of the primary disease constitute crucial measures for improving patient prognosis.

The exact pathogenic mechanisms of PG remain incompletely elucidated. Its etiology is multifactorial, encompassing genetic predisposition, physical trauma, pharmacologic triggers, and coexisting systemic diseases (12). These factors are believed to interact with dysregulated immune responses and abnormal activation of inflammatory signaling pathways, leading to neutrophil-driven inflammatory damage (29, 30). Of note, studies have proposed that a hyperinflammatory response to *Escherichia coli* may contribute to the inflammatory processes of IBD and its cutaneous manifestation of PG, implying that persistent inflammation resulting from dysbiosis of colonizing bacteria may also represent a potential pathological mechanism (31). In this case, repeated surgical debridement likely complicated PG management by impairing wound healing, promoting local bacterial colonization, and potentially exacerbating inflammation.

To date, no standardized treatment guidelines have been established for PG. Systemic CS administered at a daily dose of 0.5–1 mg/kg remains the cornerstone of therapy; however, only 32% of patients achieve complete or partial healing (32, 33). Furthermore, PG cases complicated by underlying HMs, such as AML, typically exhibit favorable responses to chemotherapy (34). Our patient experienced the recurrence of PG during CS dose tapering, which coincided with the progression of the underlying HM, suggesting that the activity of ND is closely associated with the status of the primary disorder. This underscores the importance of clinicians to maintain vigilance regarding the disease activity of PG-inducing underlying conditions (34).

The patient continues to receive MDS-targeted therapy and has maintained a consistently optimistic attitude. The protracted and intricate diagnostic and therapeutic course of this case offers several important lessons. First, a high degree of vigilance for NDs is essential in clinical practice. In particular, misdiagnosis of PG or inappropriate surgical debridement may accelerate tissue ulceration and necrosis, owing to the pathergy phenomenon unique to this condition. Second, for cases initially suspected of SSTI, if pathological examinations reveal skin lesions dominated by neutrophilic infiltration, accompanied by negative microbial test results and poor response to anti-infective therapy, the scope of differential diagnosis should be expanded to facilitate timely definitive diagnosis. Third, NDs are often associated with underlying systemic disorders, and their onset may follow, precede, or be concomitant with the ND’s diagnosis. When NDs precede the onset of the underlying disease, diagnostic delay is far more likely. Therefore, systematic screening should be prioritized for underlying disorders in all ND cases, given that NDs may serve as a potential early indicator of hematological disorders. In such contexts, early diagnosis and targeted treatment are critical for optimizing patient’s survival.

## Conclusion

This report underscores an unusual and diagnostically challenging case of PG, in which the patient presented with manifestations mimicking an SSTI several months prior to the definitive diagnosis of MDS. We recommend that clinicians promptly conduct a comprehensive diagnostic evaluation for patients presenting with neutrophil-predominant cutaneous lesions if surgical debridement or broad-spectrum antibiotic therapy fails to alleviate symptoms and if microbiological investigations yield negative results. Furthermore, in the rare instances where NDs are diagnosed initially, a thorough workup should be undertaken for underlying hematologic disorders, given that cutaneous manifestations may serve as the heralding sign of an occult systemic disease.

## Data Availability

The original contributions presented in the study are included in the article/supplementary material, further inquiries can be directed to the corresponding author.
